# A novel family of defensin-like peptides from *Hermetia illucens* with antibacterial properties

**DOI:** 10.1186/s12866-024-03325-1

**Published:** 2024-05-16

**Authors:** Leila Fahmy, Tomas Generalovic, Youssif M. Ali, David Seilly, Kesavan Sivanesan, Lajos Kalmar, Miha Pipan, Graham Christie, Andrew J Grant

**Affiliations:** 1 Department of Veterinary Medicine, University of Cambridge, Cambridge, UK; 2 Better Origin, Future Business Centre, Cambridge, UK; 3 Department of Zoology, University of Cambridge, Cambridge, UK; 4 Department of Chemical Engineering and Biotechnology, University of Cambridge, Cambridge, UK

**Keywords:** AlphaFold, Antimicrobial peptides, Antibiotics, Black soldier fly larvae, Defensins, *Hermetia illucens*, *Pseudomonas aeruginosa*

## Abstract

**Background:**

The world faces a major infectious disease challenge. Interest in the discovery, design, or development of antimicrobial peptides (AMPs) as an alternative approach for the treatment of bacterial infections has increased. Insects are a good source of AMPs which are the main effector molecules of their innate immune system. Black Soldier Fly Larvae (BSFL) are being developed for large-scale rearing for food sustainability, waste reduction and as sustainable animal and fish feed. Bioinformatic studies have suggested that BSFL have the largest number of AMPs identified in insects. However, most AMPs identified in BSF have not yet undergone antimicrobial evaluation but are promising leads to treat critical infections.

**Results:**

Jg7197.t1, Jg7902.t1 and Jg7904.t1 were expressed into the haemolymph of larvae following infection with *Salmonella enterica* serovar Typhimurium and were predicted to be AMPs using the computational tool ampir. The genes encoding these proteins were within 2 distinct clusters in chromosome 1 of the BSF genome. Following removal of signal peptides, predicted structures of the mature proteins were superimposed, highlighting a high degree of structural conservation. The 3 AMPs share primary sequences with proteins that contain a Kunitz-binding domain; characterised for inhibitory action against proteases, and antimicrobial activities. An in vitro antimicrobial screen indicated that heterologously expressed SUMO-Jg7197.t1 and SUMO-Jg7902.t1 did not show activity against 12 bacterial strains. While recombinant SUMO-Jg7904.t1 had antimicrobial activity against a range of Gram-negative and Gram-positive bacteria, including the serious pathogen *Pseudomonas aeruginosa*.

**Conclusions:**

We have cloned and purified putative AMPs from BSFL and performed initial in vitro experiments to evaluate their antimicrobial activity. In doing so, we have identified a putative novel defensin-like AMP, Jg7904.t1, encoded in a paralogous gene cluster, with antimicrobial activity against *P. aeruginosa.*

**Supplementary Information:**

The online version contains supplementary material available at 10.1186/s12866-024-03325-1.

## Background

The world faces a major infectious disease challenge, exacerbated by growing levels of antimicrobial resistance (AMR). New therapeutic modalities are desperately needed, with increasing attention being paid to the potential of antimicrobial peptides (AMPs) [1, 2].

AMPs are typically small proteins that are usually formed from fewer than 50 amino acids and possess molecular weights (MWs) of around 10 kDa. AMPs are stable and retain activity across a wide pH range [3]. Many are cationic, with membrane interactions mediated by electrostatic forces between positively charged AMPs and negatively charged bacterial membranes and with other negatively charged structures, e.g., lipopolysaccharide, lipoteichoic acid and DNA [4]. Interactions between AMPs and eukaryotic cells are weak compared to bacterial membranes, as the outer layer of eukaryotic cells is composed of zwitterionic phosphatidylcholine and sphingomyelin that display neutral charge at physiological pH [4, 5]. Anionic AMPs use environmental metal ions to form salt bridges that facilitate bacterial membrane disruption [6]. AMPs demonstrate a broad-spectrum of antimicrobial actions against a range of microorganisms including bacteria, fungi, viruses, and other microbes [7].

AMPs can be divided into 4 categories based on their structural elements and can be classed as either linear α-helical peptides, β-sheet containing peptides, linear extended peptides that are devoid of α- or β-structures, or they can be classed as peptides containing a mix of α- and β-elements [8]. Although these structural similarities can be used to classify AMPs, peptides with even minor differences in physicochemical and structural properties may have significantly different activities [9]. Therefore, AMPs have been further classified into families which consider features such as structure, amino acid composition, and source organism, which helps to better discriminate collections of AMPs. Defensins are the most well studied family of AMPs and are primarily defined structurally through their *N*-terminal loop followed by an antiparallel β-sheet core that is stabilised through 3 to 4 intramolecular disulphide bonds [8]. Based on the spatial distribution of cysteines, defensins are further subdivided into α-, β- and θ-defensins [8]. Defensins are small (2–6 kDa) AMPs that are typically rich in basic arginine and lysine residues which provide the AMPs with an overall cationic nature [10]. Defensins are mostly active against Gram-positive bacteria, but some also display antimicrobial activity against Gram-negative bacteria and fungi [11].

The mechanisms of antimicrobial action of AMPs are highly diverse. The most investigated mechanism of AMPs is their ability to disrupt membranes [12]. Membrane-acting AMPs lead to peptide-peptide or lipid-peptide complexes; when a critical concentration is reached, AMPs penetrate the hydrophobic core of the bilayer, and form transmembrane pores and cytoplasmic membrane lysis through models such as toroidal pore, carpet-like and barrel stave [1, 5, 13, 14]. Non-membrane-acting AMPs translocate across membranes without damage, destabilising cell function by direct interaction with DNA, RNA, and protein [4, 13]. Some AMPs can interact with various membrane and non-membrane based targets in different bacterial species [15], for example, certain AMPs may kill one species of bacteria through membrane disruption whilst they may target intracellular structures against another species .

AMPs act on bacteria quickly and have a low risk of generating resistance as it is difficult for bacteria to redesign membranes with resistance to disruption [16–18]. Rapid bactericidal activity reduces treatment duration; fewer bacterial generations lower the potential for resistance development [1, 19]. The short half-life of AMPs contributes to low environmental persistence, and their bactericidal effect is independent of growing state, whereas conventional antibiotics kill dividing cells [1, 19].

Insects AMPs are the main effector molecules of their innate immune system, produced locally at different surface epithelia or secreted systemically by haemocytes and fat bodies into haemolymph when triggered by pathogen recognition [13, 14, 20]. They are generally unexplored for drug development and other applications [21], with the most studied being cecropins, defensins and attacins.

The greater wax moth, *Galleria mellonella*, and the fruit fly, *Drosophilia melanogaster*, have traditionally been used to investigate insect immune responses. However, recently, the immune system of the Black Soldier Fly (BSF), *Hermetia illucens* (L.) (Diptera: Stratiomyidae), has attracted attention [22].

*H. illucens* is a non-pest synanthropic species native to the neotropics and common in regions with temperate climates [23]. The larvae reside in large colonies that feed on decaying material such as animal and plant wastes [24]. The species is of growing interest in industrial and research sectors engaged in the bioeconomical production of food, the bioremedial management of waste, and the development of novel therapeutics [25–27].

*H. illucens* have the largest number of AMPs identified in insects, some of which can be induced by feeding on a diet with bacteria or using different organic substrates [14, 28, 29]. Bioinformatic analysis of the BSFL genome has identified 57 putatively active AMPs [13]. To date, 17 AMPs from *H. illucens* have been characterised for their in vitro antimicrobial activity against bacteria [21, 25, 30–36]. Hidefensin-1 and Hidiptericin-1 inhibit the growth of *Streptococcus pneumoniae* and *Escherichia coli*, while HiCG13551 inhibits the growth of *S. aureus* and *E. coli* [21]. Hill-Cec1 and Hill-Cec10 are bactericidal, with membrane permeabilising effects against Gram-negative bacteria, including *Klebsiella pneumoniae* and multidrug resistant (MDR) *Pseudomonas aeruginosa*, and can prevent *Pseudomonas aeruginosa* biofilm formation [18, 37]. However, most AMPs identified in BSF have not yet undergone antimicrobial evaluation but are promising leads to treat infections [20].

The aim of this study was to identify and produce novel AMPs from *H. illucens*, to enhance our repertoire of antimicrobial options. To achieve this aim, we identified novel putative AMPs from *H. illucens* through studies of secreted proteins in the larval haemolymph and genomic analyses. We then prepared and purified recombinant putative AMPs, and subsequently phenotypically characterised them against various species of bacteria including bacterial pathogens presenting a risk to public health.

## Results

### Identification of 3 putative AMPs from *H. Illucens*

Fifth instar *H. illucens* larvae were infected with *Salmonella enterica* serovar Typhimurium strain SL1344 to stimulate the expression of AMPs. Haemolymph of the infected larvae was harvested to collect the AMPs that had been secreted to tackle the infection. Low molecular weight proteins, such as typical AMPs, that were in the harvested haemolymph were selected for using the flow through of ultra-centrifugal filters with a molecular weight cut-off of 10 kDa. The low molecular weight proteins were resolved using SDS-PAGE and detected using silver staining (Supplementary Fig. 1). Bands were excised and underwent liquid chromatography tandem mass spectrometry (LC-MS/MS) to identify fragment protein sequences which were scanned against the translated coding sequences (CDSs) of the BSF genome. This process identified CDSs of genes expressed in larvae that had been infected with *S.* Typhimurium.

The translated CDSs of the BSF genome (GenBank: GCA_905115235.1, BioSample: SAMEA6847289.) [38] were computationally scored for likelihood of encoding an AMP. Three CDSs that scored highly using the ampir index of AMP prediction [39], were identified to also be contained in the haemolymph of larvae infected with *S.* Typhimurium SL1344 (Table 1). Jg7197.t1 (ampir score = 0.991), Jg7902.1 (ampir score = 0.961) and Jg7904.t1 (ampir score = 0.952) were each between 77 and 79 amino acids in length with molecular weights between 8.3 and 8.9 kDa. All 3 CDSs encoded proteins with signal peptides between 19 and 21 amino acids, which is a feature indicative of secreted proteins. Jg7197.t1 and Jg7902.t1 possessed pIs near 8 and as a result were predicted to be cationic at pH 7.0, with theoretical charges of + 1.72 and + 2.63, respectively. Using the Eisenberg scale, all proteins were calculated to be hydrophobic which is indicative of membrane disruptive ability that underpins the classical mechanism for AMP functionality [40, 41]. Of the 17,664 CDSs analysed using ampir from the BSF genome, Jg7197.t1, Jg7902.t1 and Jg7904.t1 ranked as the 8th, 57th and 71st CDSs most likely to encode AMPs, respectively.

**Table 1:** Jg7197.t1 Jg7902.t1 and Jg7904.t1 were detected in the haemolymph of BSF larvae exposed to an *S*. Typhimurium infection and were computationally predicted to be AMPs

Protein	Coding sequence (signal peptide underlined)	ampir index (< 1)	Rank	Amino acids	MW of pro-peptide (kDa)	Charge (pH 7.0)	pI	Hydrophobicity
Jg7197.t1	*MKVFLLILLLISIIATAFS*GSINRDMCSQPAQAGRCFATMERYHYKSDINECMKFIYGGCGGNNNNFMTREACEVACKV	0.991	8th	79	8.87	+ 1.72	8.11	0.17
Jg7902.t1	*MRFMLAVFVLISLFAAILA*AGNPVCSLPKDVGPCRAGKPRFFYNTATKQCERFMYGGCQGNENNFETIDACKAACSN	0.961	57th	77	8.39	+ 2.63	8.38	0.16
Jg7904.t1	*MKLSVSFLFVLCCFLSVVVA*RNEDVCSQPLIIGTCRARIPLYYFDSKTNSCEKFEYGGCDGNDNQFATLDECKKACM	0.952	71st	77	8.65	-1.49	4.92	0.16

### *In silico* characterisation of 3 putative AMPs from *H. Illucens*

**Fig. 1:**
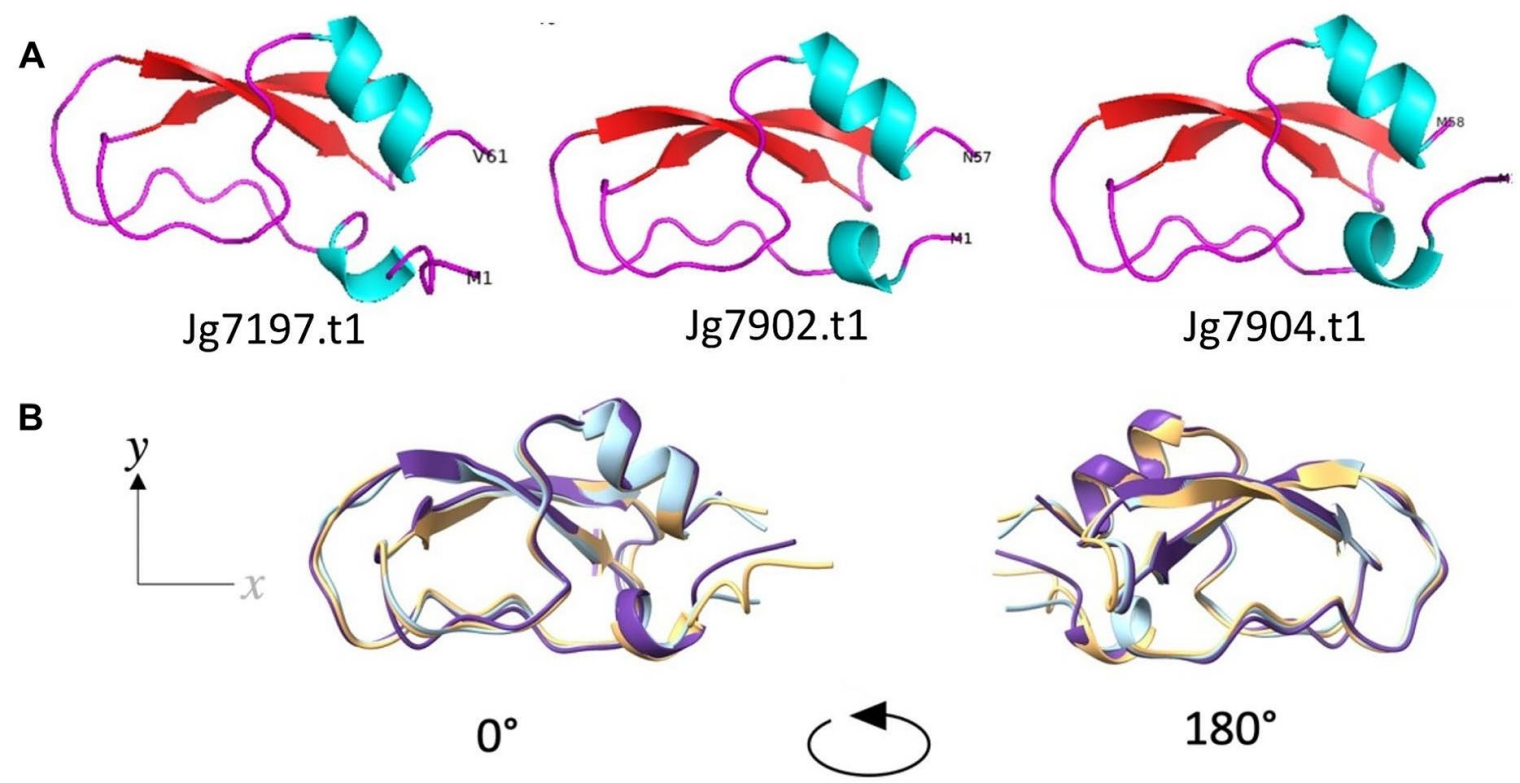
Structure predictions of the predicted AMPs; Jg7197.t1, Jg7902.t1, and Jg7904.t1. (**A)** The tertiary structures of the 3 proteins were predicted using AF2 and the structures were visualised using PyMol. Helical structures are coloured blue; looped regions are coloured magenta and sheets are coloured in red. The 3 proteins held an αβ-structural motif. (**B)** Superimposition of the 3 AF2 models demonstrated the high degree of similarity between the protein structures. Jg7197.t1 is shown in peach, Jg7902.t1 is shown in light blue and Jg7904.t1 is shown in purple

Model structures of the mature proteins without signal peptides were predicted using AlphaFold v2.1.0 (AF2) [42] (Fig. 1A). All predicted structures shared an αβ-motif composed of 2 antiparallel β-strands flanked by short a-helices. Superimposition of the proteins highlighted the high degree of similarity between each of the predicted structures (Fig. 1B). There were high confidences in the structure predictions (Supplementary Fig. 2A). The cysteine rich αβ-motif model structures were likely stabilised through disulphide bonds, as all 3 structures held pairs of cysteine residues within 7.5Å of each other (Supplementary Fig. 2B), which has been identified as a distance limit for disulphide bond formation [43]. Cysteine-stabilised αβ-motif structures are typical of the defensin family of AMPs [10].

The 3 predicted AMPs shared primary protein sequences with proteins containing a Kunitz-binding domain and several toxins (Supplementary Table 1).

### Genomic clustering of the novel putative AMPs

The genes of the 3 putative AMPs were found to lie within 2 distinct paralogous clusters of short gene sequences in chromosome 1 of the *H. illucens* genome. Jg7197.t1 was in a gene cluster of 5 short gene sequences, whilst Jg7902.t1 and Jg7904.t1 were both contained in a cluster of 4 short paralogous gene sequences. The CDSs contained within these gene clusters were all predicted to likely encode AMPs (Table 2). Like the representative sequences, Jg7197.t1, Jg7902.t1 and Jg7904.t1, all sequences within the paralogous gene clusters were found to contain signal peptides between 18 and 22 amino acids and mature protein sequences ranged between 56 and 60 amino acids, with molecular weights between 6.4 and 7.0 kDa. However, despite these similarities the paralogous proteins differed by theoretical pIs, resulting in changes to surface charges. This may reflect a functional divergence or be indicative of the environment in which the proteins are secreted.

**Table 2:** Paralogous gene clusters encoding predicted AMPs including Jg7197.t1, Jg7902.t1 and Jg7904.t1

Paralogous gene cluster	Gene	ampir score	MW (kDa) of mature protein	pI	Charge at pH 7.0
1	*jg7201.t1*	0.988	6.53	5.194	-2.76
	*jg7200.t1*	0.920	6.58	5.269	-2.69
	*jg7199.t1*	0.903	6.92	6.167	-0.68
	*jg7198.t1*	0.828	6.74	6.234	-0.53
	*jg7197.t1*	0.991	6.78	7.344	+ 0.57
2	*jg7901.t1*	0.979	6.92	7.187	+ 0.30
	*jg7902.t1*	0.961	6.16	7.684	+ 1.33
	*jg7903.t1*	0.982	6.61	6.359	-0.86
	*jg7904.t1*	0.952	6.46	4.821	-2.93

**Fig. 2:**
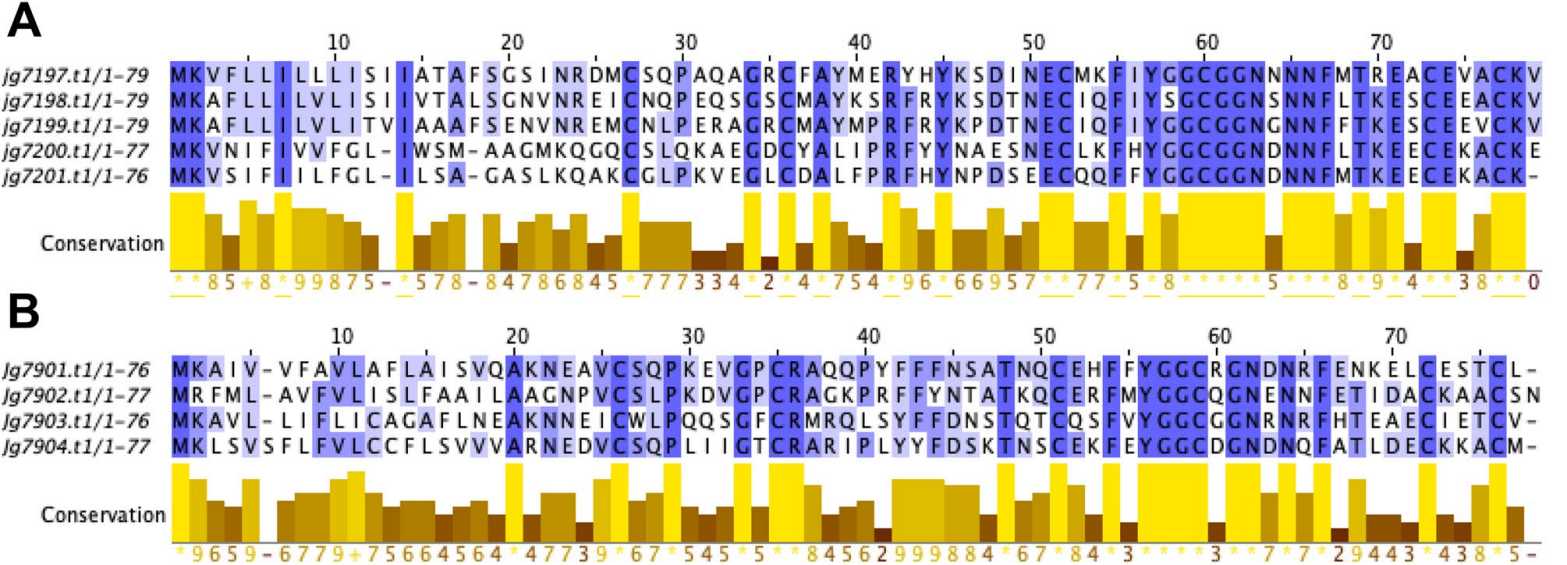
Multi-sequence alignments of paralogous gene cluster protein sequences. (**A**) Sequence alignments of the protein sequences encoded by the gene *jg719.t1* cluster and (**B**) the *jg7902.t1* and *jg7904.t1* gene cluster. Sequences are coloured dark blue to show high conservation in residues and light blue to show low conservation. Multisequence alignment was performed with TCoffee and visualised with Jalview

Multi-sequence alignments of the 2 paralogous gene clusters revealed a high degree of sequence conservation (Fig. 2). The greatest conservation was seen at the *C*-termini of the paralogous clusters whilst the *N*-terminal signal peptide sequences were less well conserved. In both clusters, 6 cysteines were conserved, therefore the formation of 3 disulphide bonds is likely shared between all paralogous proteins. The same structural feature is strongly conserved within the defensin family of AMPs [10].

**Fig. 3:**
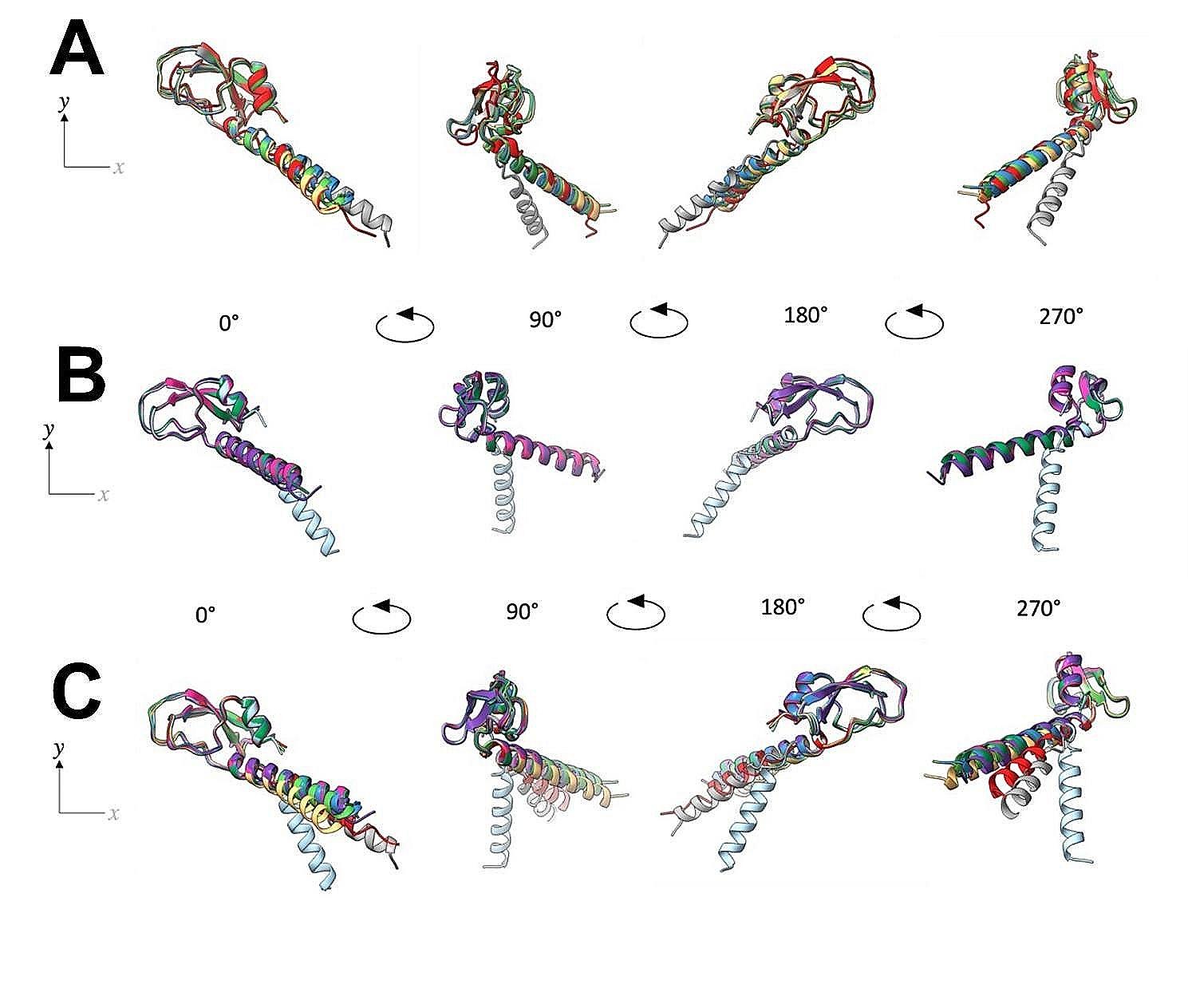
Superimposition of the predicted tertiary structures of the proteins encoded within the novel putative AMP gene clusters. (**A**) Predicted tertiary structures of proteins encoded by the *jg7197.t1-jg7201.t1* gene cluster, and (**B**) the *jg7901.t1-jg7904.t1* gene cluster. (**C**) Structures from both sets of gene clusters were also overlain to demonstrate that despite the genomic separation between the 2 gene clusters, the structures were highly conserved. In the *jg7197.t1-jg7201.t1* gene cluster; Jg7197.t1 is yellow, Jg7198.t1 is light green, Jg7199.t1 is dark blue, Jg7200.t1 is red and Jg7201.t1 is grey. In the *jg7901.t1-jg7904.t1* gene cluster; Jg7901.t1 is pink, Jg7902.t1 is light blue, Jg7903.t1 is dark green and Jg790.t14 is purple

The structures of the pro-peptide proteins encoded by the paralogous gene clusters were predicted in AF2. All proteins in both paralogous gene clusters held the highly conserved αβ-motif structure seen in Jg7197.t1, Jg7902.t1 and Jg7904.t1 (Fig. 3A and B). Interestingly, all structures contained an *N*-terminal α-helical signal peptide with different kink-angles between each of the structures. Despite the gene clusters being in distinct regions, separate from one another on the genome, the structures were highly conserved between the 2 clusters (Fig. 3C).

### Heterologous expression of recombinant putative AMPs

**Fig. 4:**
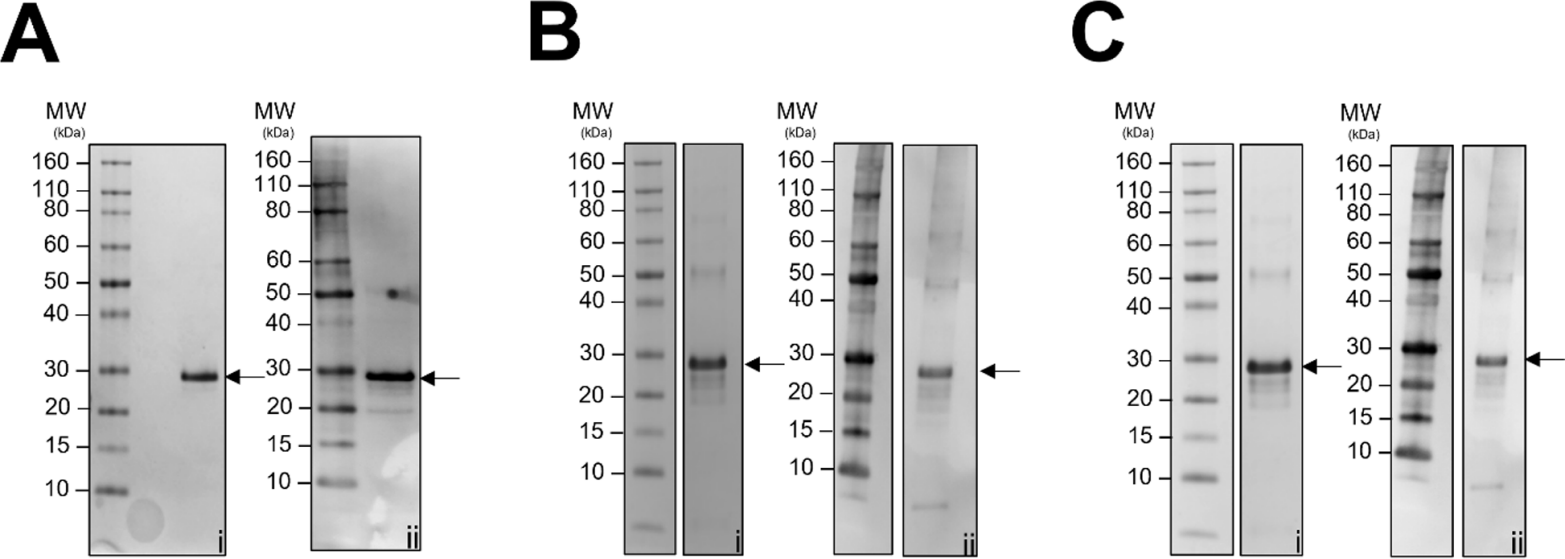
Heterologous expression of recombinant putative AMPs. Recombinant protein constructs containing *N*-terminal 6xHis and SUMO tags of (**A**) Jg7197.t1 (23.5 kDa), (**B**) Jg7902.t1 (23.5 kDa) and (**C**) Jg7904.t1 (23.8 kDa) were expressed and purified. The recombinant proteins were detected using SDS-PAGE (i), and the presence of the 6xHis tag was confirmed through western blot (ii). Full length gels and blots are shown in Supplementary Fig. 4

The representative putative AMPs, Jg7197.t1, Jg7902.t1 and Jg7904.t1, identified in the haemolymph of larvae infected with *S.* Typhimurium and computationally predicted to be AMPs, were produced as recombinant proteins. The 3 sequences were expressed in *E. coli* BL21 (DE3) as constructs containing an *N*-terminal 6xHis tag, and SUMO solubility tag. Despite the SUMO tag, all 3 proteins were expressed mostly as insoluble inclusion bodies (Supplementary Fig. 3). To purify from inclusions, the recombinant proteins were isolated from bacterial cultures that had been induced with 0.5mM IPTG. A purification approach was taken to chemically solubilise the recombinant proteins and capture them through nickel affinity chromatography. The solubilisation agent was then gently removed from the protein environment to allow for refolding. Molecular weights of the recombinant fusion proteins were 23.5 kDa, 23.5 kDa and 23.8 kDa for Jg7197.t1, Jg7902.t1 and Jg7904.t1, respectively. SDS-PAGE and western blotting confirmed the presence of the purified recombinant proteins (Fig. 4A-C, Supplementary Fig. 4).

### Phenotypic characterisation of the putative AMPs

The recombinant proteins were screened for antimicrobial activity against a panel of 12 strains of bacteria including representative Gram-negative and Gram-positive species. The in vitro assay periodically measured ODs of bacterial cultures exposed to 250 µg/mL recombinant protein in a 96-well plate format over the course of 8 h. Growth curves for each assay were generated and area under the curve (AUC) was used to determine statistical significance of exposure to the recombinant protein. As the recombinant proteins were in a buffer containing 500mM Tris pH 8.0, a control of each bacterial culture grown in the buffer alone was also included to determine if any effect observed was because of the buffer or the protein. An undisturbed growth control was also included, consisting of bacteria grown in Mueller Hinton Broth media without the addition of protein or buffer.

**Fig. 5:**
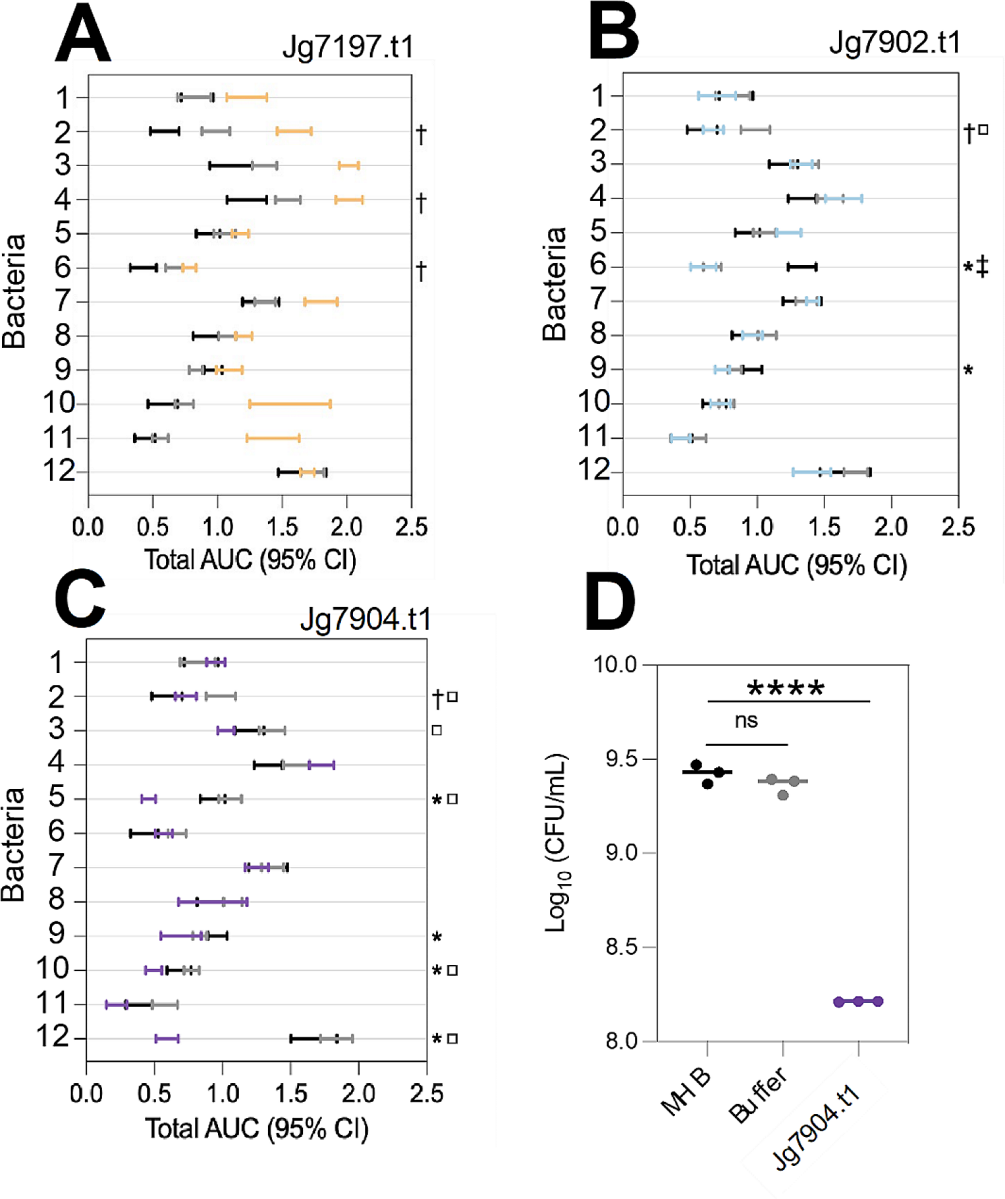
Antimicrobial activity of recombinant Jg7197.t1, Jg7902.t1 and Jg7904.t1. AUCs of OD measurements of bacterial cultures exposed to 250 µg/ml of (**A**) Jg7197.t1, (**B**) Jg7902.t1 and (**C**) Jg7904.t1. * = indicates when the bacteria exposed to the recombinant protein were reduced compared to the growth control population. □ = indicates when the bacteria exposed to the recombinant protein were reduced compared to the buffer population. † = indicates when the growth control population of bacteria was reduced compared to the bacteria exposed to the buffer. Populations labelled with the combination of * □ were reduced in size due to the direct effect of the recombinant protein. (**D**) Total number of viable cells were measured as CFUs in *P. aeruginosa* PaO1 cultures exposed to Jg7904.t1 (500 µg/ml, 21.0µM; purple) for 8 h and compared with undisturbed growth (black), and buffer control (grey) populations. 1 = *Staphylococcus aureus* ATCC 10,788, 2 = *S. aureus* LGA251, 3 = *S*. Typhimurium SL1344, 4 = *Salmonella enterica* serovar Enteritidis NCTC 13,349, 5 = *P. aeruginosa* PaO1, 6 = *Listeria monocytogenes* EGD-e, 7 = *Klebsiella pneumoniae* 43,816, 8 = *Escherichia coli* K12, 9 = *Bacillus thuringiensis* serovar finitimus TBt020, 10 = *Bacillus subtilis* 168, 11 = *Bacillus megaterium* QM B1551, 12 = *Bacillus cereus* ATCC 14,579. Statistics were performed using an unpaired Students’ t-test: ns (not significant), **** *p* ≤ 0.0001

Recombinant Jg7197.t1 (250 µg/ml, 9.88µM) did not demonstrate any clear antimicrobial activity in the assay (Fig. 5A). Similarly, recombinant Jg7902.t1 (250 µg/ml, 10.64µM) also did not demonstrate a clear inhibitory phenotype against the 12 strains of bacteria used in the assay (Fig. 5B).

Contrastingly, recombinant Jg7904.t1 demonstrated antimicrobial activity against both Gram-negative and Gram-positive bacteria (Fig. 5C). Over the course of the 8-hour assay, recombinant Jg7904.t1 (250 µg/ml, 10.5µM) inhibited the growth of the Gram-negative pathogen *P. aeruginosa* PaO1 as well as the Gram-positive species *Bacillus megaterium* QM B1551 and *Bacillus cereus* ATCC 14,579.

To confirm the antimicrobial activity of recombinant Jg7904.t1 against the ESKAPE pathogen *P. aeruginosa*, the number of viable cells following the 8-hour assay was determined through CFU measurement (Fig. 5D). Incubation of *P. aeruginosa* PaO1 cultures with SUMO-Jg7904.t1 (500 µg/mL, 21.0µM) significantly reduced the number of viable *P. aeruginosa* PaO1 cells compared to the growth control (*p* ≤ 0.0001). Whilst the buffer control (containing 50mM Tris pH 8.0) did not demonstrate any effect on the number of viable cells in the population, confirming that the antimicrobial action was a result of Jg7904.t1.

## Discussion

In this study we took a comprehensive approach to identify BSF AMPs which combined bacterial challenge of larvae, proteomics, *in silico* analyses, recombinant protein engineering and in vitro antimicrobial studies. We aimed to stimulate AMP expression in 5th instar *H. illucens* larvae through infection with *S*. Typhimurium SL1344. Subsequently, small proteins within the haemolymph were identified through LC-MS/MS. Using the machine learning tool ampir, translated gene sequences were scored on likelihood of encoding AMPs. Three sequences that had scored highly with ampir were also identified through LC-MS/MS to have been secreted into the larval haemolymph. The 3 sequences were identified in LC-MS/MS datasets of larvae that had been infected with *S*. Typhimurium SL1344. The proteins may be secreted in response to infection or expressed constitutively, or they may have been expressed due to injury from the handling and injection processes. To help select candidate sequences from the long list of putative AMPs generated by ampir, the LC-MS/MS experimental dataset confirmed 3 protein sequences that were highly likely to be AMPs (ampir score > 0.9) and were also secreted into the haemolymph of 5th instar larvae following handling and injection. Following, identification of candidate AMP sequences a recombinant protein engineering approach was taken to produce the Jg7197.t1, Jg7902.t1 and Jg7904.t1 in *E. coli* which were subsequently tested using an in vitro antimicrobial screening assay.

Other discovery platforms for the identification and production of AMPs include isolation of native AMPs from haemolymph and concentrating through processes such as organic solvent precipitation or chromatography techniques [24, 44]. However, these techniques require large amounts of source material to yield concentrations of AMP great enough to detect in assays or the methods may leave residual chemicals that can interfere with antimicrobial activities .

We identified that the genes of the 3 putative AMPs (*jg7197.t1, jg7902.t1* and *jg7904.t1*) were within 2 distinct paralogous clusters of short gene sequences in chromosome 1 of the *H. illucens* genome. Paralogues of gene families have been reported for insect AMPs such as the drosomycin multigene family in *Drosophila melanogaster* [45]. Such paralogous gene families, also referred to as combinatorial libraries, arise within a species through rapid evolutionary duplication events. Paralogous genes can encode protein families with functional divergence [46]. In the case of the *D. melanogaster* drosomycin paralogues, individual members of the family have been found to be expressed in specific developmental stages of the fly. Furthermore, the drosomycin paralogous genes are up-regulated in response to different stimuli and have demonstrated a range of antimicrobial activities [47]. We investigated the antimicrobial activity of 3 representatives from the family, Jg7904.t1, Jg7197.t1 and Jg7902.t1 that were selected as they had been expressed as proteins in the haemolymph of BSFL. Other members of the family were not identified in the haemolymph following microbial challenge. Within a single species, different patterns of defensin gene expression such as between tissues or life-stages have been recognised [48, 49]. As Jg7198.t1 – Jg7201.t1, Jg7901.t1 and Jg7903.t1 that were not present in the BSFL haemolymph following bacterial challenge with *S.* Typhimurium these genes may possess different patterns of expression. Future studies can explore other BSFL challenges that may stimulate AMP expression and/or which cell types these genes are expressed in.

Recent advancements in artificial intelligence and structure prediction software have provided opportunities to speed up the process of drug discovery. In this project AF2 predicted the structures of the putative AMPs Jg7197.t1, Jg7902.t1 and Jg7904.t1. In doing so, the software generated a model of atomic spatial co-ordinates that allowed for analysis of the predicted structures through other computational tools which inferred each of the proteins’ functions.

The predicted AMPs, Jg7197.t1, Jg7902.t1 and Jg7904.t1 shared primary protein sequences with proteins containing a Kunitz-binding domain which are characterised as having cysteine-stabilised αβ-motif structures [50]. Despite a high conservation in protein structure, the Kunitz-binding domain family possesses an extensively diverse functional repertoire which span proteases to antimicrobials [51]. Three isoforms of the EpTI Kunitz-binding protein, from *Erythrina poeppigiana* seeds, were found to have antimicrobial and antibiofilm activities against *Enterobacter aerogenes*, *Enterobacter cloacae*, *Klebsiella pneumoniae*, *Staphylococcus aureus* and *Staphylococcus haemolyticus* [47]. TP25 and TP26 Tissue Factor Pathway Inhibitors containing the Kunitz binding domain, from a flounder fish (*Paralichthys olivaceus*), were found to have antimicrobial activity against *Micrococcus luteus* and *S. aureus* as well as anticancer activity against the cancer cell-line HT- 29 [52]. Another Kunitz-binding domain protein from *Inga vera* seeds, IVTI, possessed bacteriostatic activity against *Escherichia coli*, fungicidal activity against *Candida buinensis*, and anticancer activity against colorectal adenocarcinoma cells [53]. The predicted AMPs also possessed similar protein sequences to several toxins, highlighting their possible role in innate immunity. These similar toxins have been evidenced to play a role in inhibition of proteases, blocking potassium channels and antimicrobial immune responses [51, 54, 55].

The signal peptide sequences of Jg7197.t1, Jg7902.t1 and Jg7904.t1 demonstrated the most variation in sequence conservation and predicted structure. Loss of sequence conservation is also shared with the drosomycin multigene family [45]. The signal peptides for the *H. illucens* Jg7197.t1, Jg7902.t1 and Jg7904.t1 were all predicted to be formed of α-helices with differing torsion angles. As signal peptides co-ordinate the secretion of the AMP from the cell it might be speculated that the genes could be expressed in different environments such as different cell types.

The putative AMPs, Jg7197.t1, Jg7902.t1 and Jg7904.t1, were produced as recombinant fusion proteins. The cysteine-rich Kunitz-domain containing protein 6xHis-SUMO-Jg7904.t1 demonstrated antimicrobial activity against *P. aeruginosa*. It should be noted, that Jg7904.t1 had a pI of 4.8 which conferred a -2.80 charge at pH 7.0. However, the recombinant protein was in a buffer at pH 8.0 which gave Jg7904.t1 a net charge of -5.08 when used in the phenotypic assays with *P. aeruginosa*. Therefore, we can expect that the Jg7904.t1 recombinant protein displayed an anionic form in the phenotypic assays. The first characterised anionic AMP was identified from the toad *Bombina maxima* and had anti-*S. aureus* activity [56]. A recent study characterised an anionic AMP with a net charge of -1 that was isolated from *Moringa oleifera* seeds and had antimicrobial anti-*S. aureus* activity [57]. Anionic AMPs require divalent cations as functional cofactors. The cations form cationic salt bridges between the anionic bacterial outer membrane and in the anionic AMP [58, 59]. Daptomycin, a clinically relevant anionic lipopeptide AMP, requires Ca^2+^ to interact with membranes [60]. The human anionic AMP, dermcidin, DCD-1 L, adopts a random coil structure in aqueous environments. Upon interaction with a membrane, DCD-1 L folds into an α-helical conformation that oligomerises into complexes stabilised with Zn^2+^ [6]. In addition to membrane activity, anionic AMPs have also been shown to interact with intracellular processes including inhibition of ribonucleases [60]. Further studies are required to assess the effect of different cations as functional cofactors on the antimicrobial activity of Jg7904.t1, as well as Jg7197.t1 and Jg7902.t1.

In the phenotypic screen used, 6xHis-SUMO-Jg7904.t1 possessed antimicrobial activity against *P. aeruginosa*, however, no antimicrobial activities were measured in OD analyses of 6xHis-SUMO-Jg7197.t1 and 6xHis-SUMO-Jg7902.t1 against a panel of 12 bacterial strains. The assays performed in this study were against a limited range of microbial species which only included strains of bacteria. There is a possibility that Jg7197.t1 and Jg7902.t1 could possess antimicrobial activities against other species of bacteria, and/or they may possess antiviral, antifungal or antiparasitic activity. Functional diversity within AMP families is result of evolutionary changes caused by positive selection pressures [61]. As ancient effectors of innate immunity, AMPs are important players in a hosts’ first line of defence against microbial invasion [62]. Over time, AMPs have undergone an evolutionary arms race against a wide range of pathogens resulting in structural and functional diversification [62, 63]. Defensins are ubiquitous throughout Eukarya and are recognised for their ancient lineage, however, possess considerable sequence diversity and functional variation [49]. Functional diversity has been recognised within other AMP families such as the *Musca domestica* cecropin family and the human β-defensin family. Members of both families demonstrated spectras of antimicrobial activities however differed in potency and target selectivity [64, 65]. In this study, there is significant sequence diversity within the paralogous gene clusters, thus altering key protein characteristics such as pI. It could be hypothesized that Jg7197.t1 and Jg7902.t1 may be selectively antimicrobial against species or strains that were not tested in this study. Also, Jg7197.t1 and Jg7902.t1 may possess lower potencies against the panel of bacteria tested and as such their phenotypes were below the limit of detection in the assay used in this study.

Furthermore, the phenotypic assay was performed under one set of conditions. Environmental factors such as availability of salt ions and pH can alter the activities of AMPs and be optimised to improve antimicrobial activities [66]. In addition, this study focused on 12 species/strains of representative Gram-positive and Gram-negative bacteria, therefore there may be other species for which the AMPs could show activity and the inclusion of just one strain per species limits conclusions for activity against the species. Future studies to characterise the members of the *H. illucens* gene clusters could explore optimisation of screening conditions and testing against more bacterial species and multiple strains for more species.

## Conclusions

There is an urgent need to develop new treatments for infections without risk of resistance [1, 2, 67]; the rapid and effective antimicrobial activity of AMPs makes them promising candidates [1, 20]. In this study, we combined theoretical and experimental approaches to identify AMPs in *H. illucens* and to prepare and phenotypically test recombinant versions of these. Jg7904.t1 was shown to have antimicrobial action against *P. aeruginosa*, an opportunistic pathogen that frequently causes skin and soft tissue infections (SSTIs). AMPs are attractive therapeutic antimicrobial agents for wound healing, due to their broad-spectrum, rapid antimicrobial activity, low resistance rates, ability to achieve high drug concentrations at the target, and the ability of some to promote wound healing. Therefore, Jg7904.t1 may be a promising candidate for the treatment of bacterial SSTI and wound infections caused by *P. aeruginosa*. Future studies will be required to further investigate the antimicrobial activity of the AMPs by examining their inhibitory and killing activities against different bacterial species and strain, for example ESKAPE bacterial pathogens. It will be necessary to investigate the mechanism of action by characterising bacterial time-to-kill kinetics, stability in physiological conditions, interactions with bacterial membranes, effect on bacterial morphology and anti-biofilm activity. It will also be necessary to investigate haemolytic activity (destroying red blood cells) and toxicity against eukaryotic cells as well as determining stability. Moreover, there has been recent interest in the use of *H. illucens* in the bioeconomy for bio-based processes and products e.g., food sustainability, waste reduction and as sustainable animal and fish feed [20, 23, 67]. We contend that *H. illucens* could be used to produce AMPs (e.g., Jg7904.t1), providing an environmental benefit from waste valorisation. Further studies will be required to establish the extraction and purification protocols to assess this and to evaluate whether this could be developed as a cost-effective method that could be scaled for pharmaceutical production.

## Methods

### Stimulating expression of AMPs in BSFL through bacterial challenge

BSFL were washed briefly with 70% ethanol and rinsed with deionised water. A needle (0.8 mm diameter) was dipped into an overnight culture of *S.* Typhimurium SL1344 (diluted to OD_600nm_ 1.5) and pricked into the body of BSFL. We chose *S*. Typhimurium as a representative bacterial species (and human pathogen) that BSFL could be exposed to in ‘real-life’ through feeding/breaking down waste food and manure. Infected BSFL were incubated for 24-hours at 30 °C with humidity. Following the day of infection, BSFL were frozen at -80 °C. To harvest haemolymph, BSFL bodies were scored using a scalpel and suspended in a tube of 1X PBS supplemented with 0.1% *N-*phenylthiourea and 1.5X protease inhibitor tablets. Tubes were centrifuged at 4 000 *x g* for 45-minutes at 4 °C to draw out the haemolymph into the suspension. To select for small biomolecules such as AMPs, the suspension was size excluded using ultra-centrifugal spin filter columns with a molecular weight cut off-of 10 kDa. The flow through was lyophilised and resuspended in 1xPBS to 20 mg/ml total protein. Protein concentration was determined using the Qubit^™^ protein assay. Protein constituents of the samples were resolved using SDS-PAGE and detected using the Pierce^™^ Silver Staining Kit following the manufacturer’s instructions. Bands were excised from the gel and analysed by LC-MS/MS. Fragment ion amino acid sequences were searched against the translated BSF genome ( [38]; genome and annotation deposited as BioSample: SAMEA6847289. Data gathered is representative of 3 biological repeats.

### Computational analysis of putative AMPs

Translated BSF coding sequences (BioSample: SAMEA6847289) detected by LC-MS/MS in BSFL challenged with *S.* Typhimuirum SL1344 were aligned using were performed using TCoffee software and visualised using Jalview [68, 69]. Signal peptides were identified using SignalP-6.0 [70]. Model structures of the mature protein sequences were predicted using the software AF2 [42], which generated Protein Databank co-ordinate files that were visualised using PyMol environment. Structural homologies were searched for using the Dali server [71] which scanned the co-ordinate files against the Protein Databank.

### Production of recombinant Jg7197.t1, Jg7902.t1 and Jg7904.t1 proteins

Jg7197.t1, Jg7902.t1 and Jg7904.t1 were identified in the haemolymph of BSFL challenged with *S.* Typhimurium SL1344. The 3 sequences were selected as representatives of the paralogous gene clusters predicted to encode novel defensin-like AMPs and were carried forward into in vitro studies. Using the pET28a(+) vector, constructs of the putative AMP genes were designed to contain N-terminal 6xHis and SUMO tags as well as a 6xHis tag at the C-terminus. Plasmids were codon optimised for *E. coli* expression and synthesized by Twist Bioscience. Plasmids were freshly transformed into *E. coli* BL21(DE3) cells and grown on LB_KAN50_ agar plates. Cultures (400 ml) were grown in 2 L baffled conical flasks at 37 °C, with 220 rpm shaking, until OD_600nm_ 0.4–0.7 was reached. Once a correct absorbance was achieved, flasks were cooled at 10 °C. Protein expression was induced with 0.5mM isopropyl-β-D-thiogalactopyranoside and cultures incubated at 10 °C for 56 h with shaking at 220 rpm. Cultures were harvested 4,000 *x g* for 20 min and pellets were resuspended in ice-cold lysis buffer (50 mM TrisHCl, pH 7.0, 150 mM NaCl and, 1.5X cOmplete^™^ protease inhibitor cocktail) then sonicated (25 kHz, 5 min total processing). Inclusion bodies were isolated from the lysate through a series of centrifugations all performed at 10,000 x *g* for 20-minutes at 4 °C. Pellets were washed in resuspension buffer (100 mM TrisHCl pH 7.0, 10 mM EDTA, 10 mM DTT, 0.5% v/v Triton X-100) and centrifuged. Pellets were resuspended and membranes and membrane proteins were solubilized in a detergent buffer (100 mM TrisHCl pH 7.0, 5 mM EDTA, 5 mM DTT, 2% v/v Triton X-100) and centrifuged. To remove nucleic acids, pellets were resuspended in a high salt buffer (100 mM TrisHCl pH 7.0, 5 mM EDTA, 5 mM DTT and, 1 M NaCl) and centrifuged and inclusion bodies were pelleted. Inclusion bodies were solubilised in a chaotropic buffer (50 mM TrisHCl pH 8.0, 8 M urea, 500 mM NaCl, 5 mM imidazole, 5 mM DTT). To capture the 6xHis tagged recombinant proteins, solubilised inclusion bodies were applied *via* syringe (5 mL/min) to the 5mL HisTrap^™^ HP column (Cytiva) and purified by nickel-affinity chromatography. Columns were washed with 10 column volumes of chaotropic buffer spiked with 20mM imidazole. Recombinant proteins were eluted with the chaotropic buffer supplemented with a gradient of imidazole (50mM – 400mM). To remove urea, eluted proteins were dialysed at 4 °C in a snakeskin tubing (12 kDa) against 750 mM arginine pH 8.0, 4 M − 500 mM urea, 5 mM CaCl_2_, 1 mM oxidised glutathione, 3 mM reduced glutathione. Urea concentration was gradually reduced from 8 M, to 4 M, to 2 M, then finally 500mM to allow for gentle refolding of the protein. Recombinant proteins were lyophilised and resuspended in ultrapure water to a concentration of 500mM Tris. Protein concentrations were determined using nanodrop. A quality control was included that ran blank buffers through the purification process, this measured the effect of the final buffer preparation in down-stream assays and is referred to as “Buffer control”.

### SDS-PAGE and western blotting

Cell lysates were analysed using SDS-PAGE and western blotting. Insoluble proteins were separated from soluble proteins through centrifugation at 16,000 *x g* for 1-minute. Whole cell lysate and fractionated samples were reduced (1X SDS, 1X reducing agent; 85 °C, 5 min) and loaded onto an 4–12% BisTris gel alongside a molecular weight ladder for reference (Novex Sharp Pre-Stained Protein Standard). Proteins were resolved through electrophoresis (200 V, 30 min) and then visualised using Coomassie blue. Western blotting confirmed the presence of 6xHis tagged proteins. Proteins were transferred from the gel onto a PVDF membrane (25 V, 1.3 A, 5-minutes) which was then blocked in (5% w/v BSA). Rabbit Anti-6xHis antibody (1:2,000, ab14923) probed the membrane and bound primary antibody was detected using the HRP-conjugated goat anti-rabbit secondary antibody (1:5,000, ab97051). HRP was detected using substrates (Supersignal PLUS West Pico).

### In vitro antimicrobial activity assay

Antimicrobial activities were defined in vitro against a panel of 12 bacterial strains: *S. aureus* ATCC 10788, *S. aureus* LGA251, *S.* Typhimurium SL1344, *S.* Enteritidis NCTC 13349, *P. aeruginosa* PaO1, *L. monocytogenes* EGD-e, *K. pneumoniae* 43816, *E. coli* K12, *B. finitimus* TBt020, *B. subtilis* 168, *B. megaterium* QM B1551, *B. cereus* ATCC 14579. Briefly, in a 96-well plate, 50 µL of overnight bacterial cultures (diluted 1:100 into fresh Mueller Hinton broth) were inoculated with recombinant protein (either 250 µg/mL or 500 µg/mL depending on batch-to-batch variation in recombinant protein purity). Wells were then topped up to a total well volume of 200 µl using Mueller Hinton broth. OD_605nm_ (Absorbance 96, Byony) and CFU measurements of wells were measured. Controls were included that measured the undisturbed growth of bacteria in Mueller Hinton broth, and the growth of bacteria in the presence of the “Buffer control” vehicle of the recombinant proteins.

### Data and statistical analysis

All statistical analyses and graphical representations were performed using GraphPad Prism, version 9 (GraphPad Software, San Diego, California USA, www.graphpad.com).

### Electronic supplementary material

Below is the link to the electronic supplementary material.

Supplementary Material 1

## Data Availability

All data generated and analysed during this study are included in this published article. The datasets used and/or analysed during the current study are available from the corresponding author on reasonable request.

## Abbreviations

AMR: Antimicrobial resistance
AMPs: Antimicrobial peptides
AF2: AlphaFold v2.1.0
BSF: Black Soldier Fly
BSFL: Black Soldier Fly Larvae
CDSs: Coding sequences
CFUs: Colony forming units
LC-MS/MS: Liquid chromatography tandem mass spectrometry
MDR: Multidrug resistant
MWs: Molecular weights
