# Supplementary material for: A novel family of defensin-like peptides from Hermetia illucens with antibacterial properties
Source: BMC Microbiol. 2024 May 16;24:167. doi: 10.1186/s12866-024-03325-1 (PMC11097590; doi:10.1186/s12866-024-03325-1)
Supplement: Supplementary file 1 — Supplementary Material 1 [file 12866_2024_3325_MOESM1_ESM.docx]

**Supplementary Information**

**
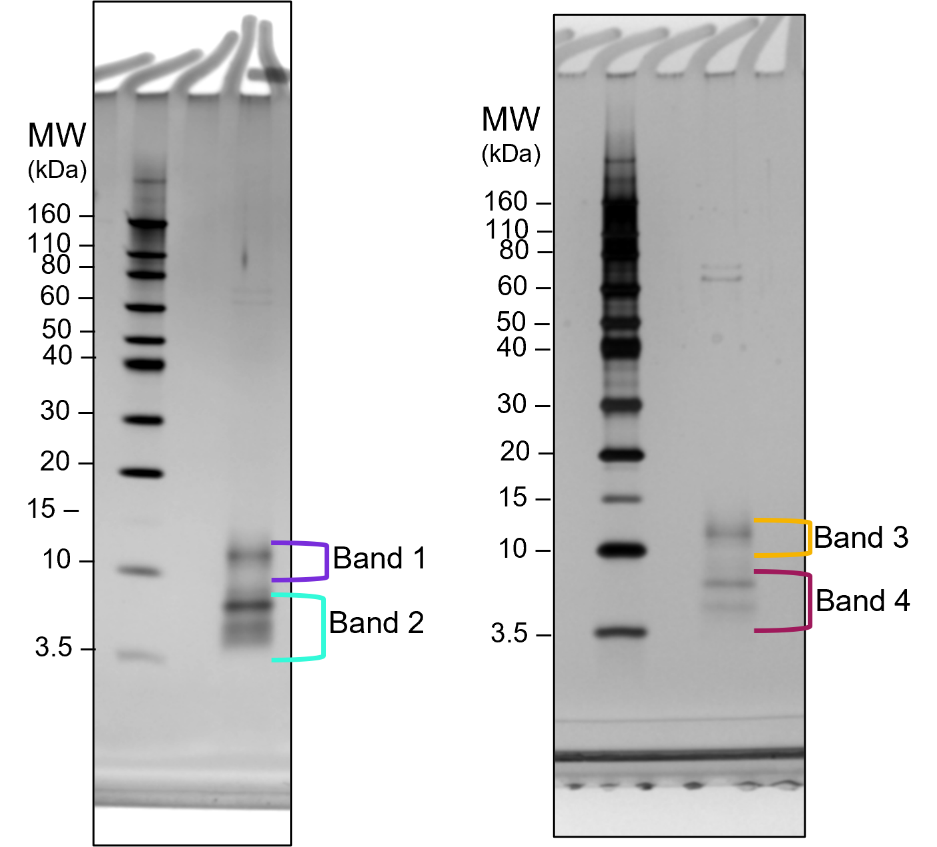
**

**Supplementary Figure 1. Two batches of larvae were exposed to *Salmonella enterica* serovar Typhimurium and possible AMPs were identified in their haemolymph.** Silver stained SDS-PAGE gel of processed larval haemolymph that had been exposed to *S*. Typhimurium infection. Bands 1, 2, 3 and 4 were excised and protein fragments were detected using LC-MS/MS. Three protein sequences were detected that also scored highly in their likelihood for being AMPs.

**Supplementary Figure 2. Predicted structure of Jg7197.t1, Jg7902.t1 and Jg7904.t1. (A)** Confidences in the AF2 model mapped as colours. Regions coloured dark blue were modelled with a high confidence and regions coloured light blue were considered good. Whilst regions coloured yellow, and orange were predicted with a lower confidence. **(B)** Each of the protein structures contained 3 pairs of cysteine residues that measured within the distance capable of forming disulphide bonds (<7.5Å).

**Supplementary Table 1. Sequence homology results of Jg7197.t1, Jg7102.t1 and Jg7904.t1.**

| **Protein** | **Description** | | **Host Species** | **Query Cover** | **E value** | **Per. ident** | **No. amino acids** |
| --- | --- | --- | --- | --- | --- | --- | --- |
| Jg7197.t1 | Pi-stichotoxin-she2a-like | XP_037903431.1 | *H. illucens* | 98% | 1e-37 | 100 | 81 |
|  | Unnamed protein product | CAD7079096.1 | *H. illucens* | 98% | 1e-37 | 100 | 79 |
|  | Pi-stichotoxin-she2a-like | XP_037903432.1 | *H. illucens* | 98% | 1e-22 | 63.33 | 79 |
|  | Unnamed protein product | CAD7079100.1 | *H. illucens* | 95% | 4e-14 | 53.45 | 76 |
|  | Papilin-like | XP_022240776.1 | *Limulus polyphemus* | 86% | 4e-14 | 57.69 | 1019 |
|  | Pi-stichotoxin-she2a-like | XP_037926239.1 | *H. illucens* | 95% | 9e-18 | 65.52 | 79 |
|  | Protease inhibitor-like | XP_017047325.1 | *Drosophila ficusphila* | 91% | 6e-14 | 51.79 | 99 |
|  | Kunitz-type serine protease inhibitor | XP_022254649.1 | *L. polyphemus* | 88% | 2e-13 | 53.70 | 198 |
|  | Boophilin | XP_037275737.1 | *Rhipicephalus microplus* | 86% | 3e-13 | 52.83 | 198 |
|  | Chelonianin-like | XP_025033037.1 | *Python bivittatus* | 91% | 3e-13 | 50 | 168 |
| Jg7902.t1 | Haemolymph trypsin inhibitor b-like | XP_037922386.1 | *H. illucens* | 96% | 4e-34 | 100 | 77 |
|  | BPTI/Kunitz domain protein | XP_037501333.1 | *Rhipicephalus sanguineus* | 96% | 1e-17 | 60.71 | 170 |
|  | Kunitz-type protease inhibitor AXPI-I | KXJ14010.1 | *Exaiptasia diaphana* | 93% | 1e-17 | 66.67 | 75 |
|  | Kappapi-actitoxin-avd3b-like | XP_043517651.1 | *Frieseomelitta varia* | 96% | 2e-17 | 60.71 | 81 |
|  | U-actitoxin-avd3j | P0DN11.1 | *Anemonia viridis* | 94% | 3e-17 | 56.36 | 82 |
|  | Papilin-like isoform X2 | XP_037499141.1 | *R. sanguineus* | 98% | 4e-17 | 62.5 | 596 |
|  | Papilin-like isoform X1 | XP_037499140.1 | *R. sanguineus* | 98% | 5e-17 | 62.5 | 596 |
|  | Kunitz-type serine protease inhibitor | XP_015282964.1 | *Gekko japonicus* | 91% | 5e-17 | 58.49 | 84 |
|  | Tissue factor pathway inhibitor-like | XP_037501030.1 | *R. sanguineus* | 98% | 5e-17 | 60.71 | 328 |
|  | Kunitz-type serine protease inhibitor 1 | GFY79245.1 | *Trichonephila madagascariensis* | 93% | 7e-17 | 57.41 | 80 |
| Jg7904.t1 | Pi-actitoxin-aeq3c-like | XP_037923343.1 | *H. illucens* | 96% | 7e-35 | 100 | 77 |
|  | Kappapi-actitoxin-avd3a | XP_034117060.1 | *Drosophila albomicans* | 91% | 5e-16 | 59.26 | 79 |
|  | Protease inhibitor-like isoform | XP_022312348.1 | *Crassostrea virginica* | 94% | 9e-15 | 55.36 | 178 |
|  | Kunitz-type protease inhibitor AXPI-I | KXJ09881.1 | *E. diaphana* | 93% | 1e-14 | 56.36 | 94 |
|  | Protease inhibitor-like isoform | XP_022312347.1 | *C. virginica* | 98% | 1e-14 | 53.45 | 218 |
|  | BPTI/Kunitz domain protein | XP_043481332.1 | *Leptopilina heterotoma* | 91% | 2e-14 | 64.15 | 139 |
|  | Protease inhibitor-like isoform | XP_022312346.1 | C. virginica | 94% | 2e-14 | 55.36 | 219 |
|  | Hypothetical protein | PAV59528.1 | *Diploscapter pachys* | 93% | 2e-14 | 54.55 | 76 |
|  | Protease inhibitor-like | XP_037922989.1 | *H. illucens* | 96% | 2e-14 | 52.63 | 76 |
|  | U-actitoxin-avd3q | P0DN18.1 | *A. viridis* | 86% | 3e-14 | 56.86 | 82 |

**
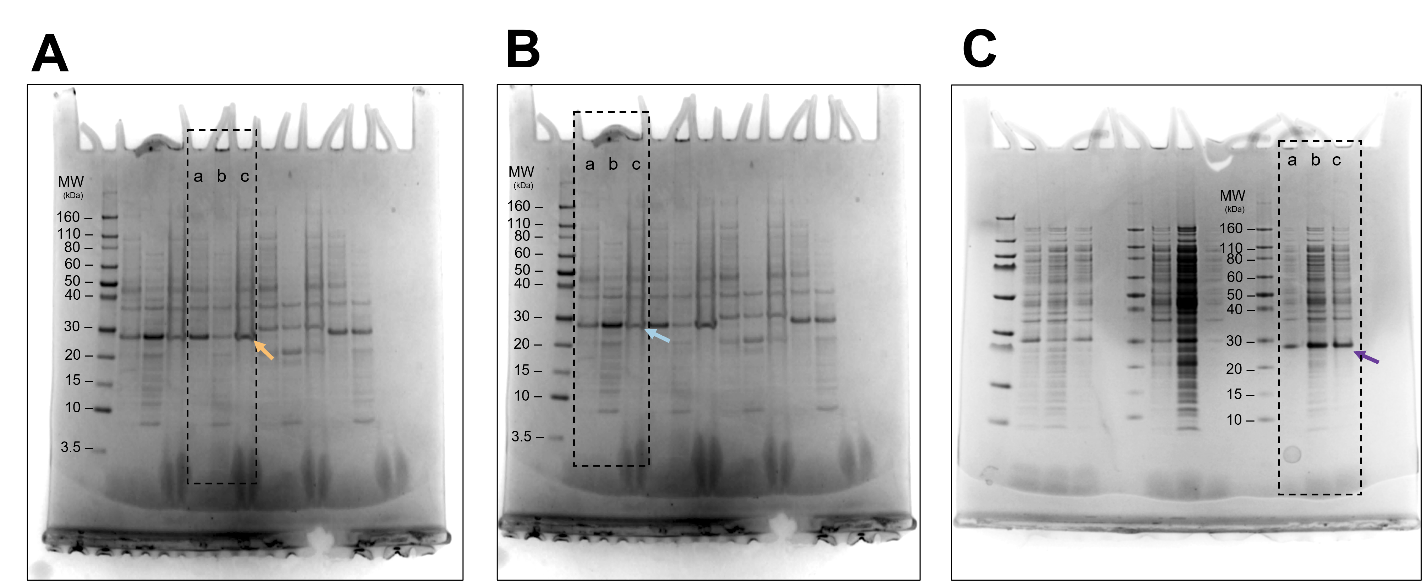
**

**Supplementary Figure 3.** **Heterologous expression of AMPs.** SDS-PAGE analysis of *E. coli* BL21 DE3 whole cell (a), soluble (b) and insoluble (c) lysates following expression of **(A)** Jg7917.t1, **(B)** Jg7902.t1 and **(C)** Jg7904.t1.

**
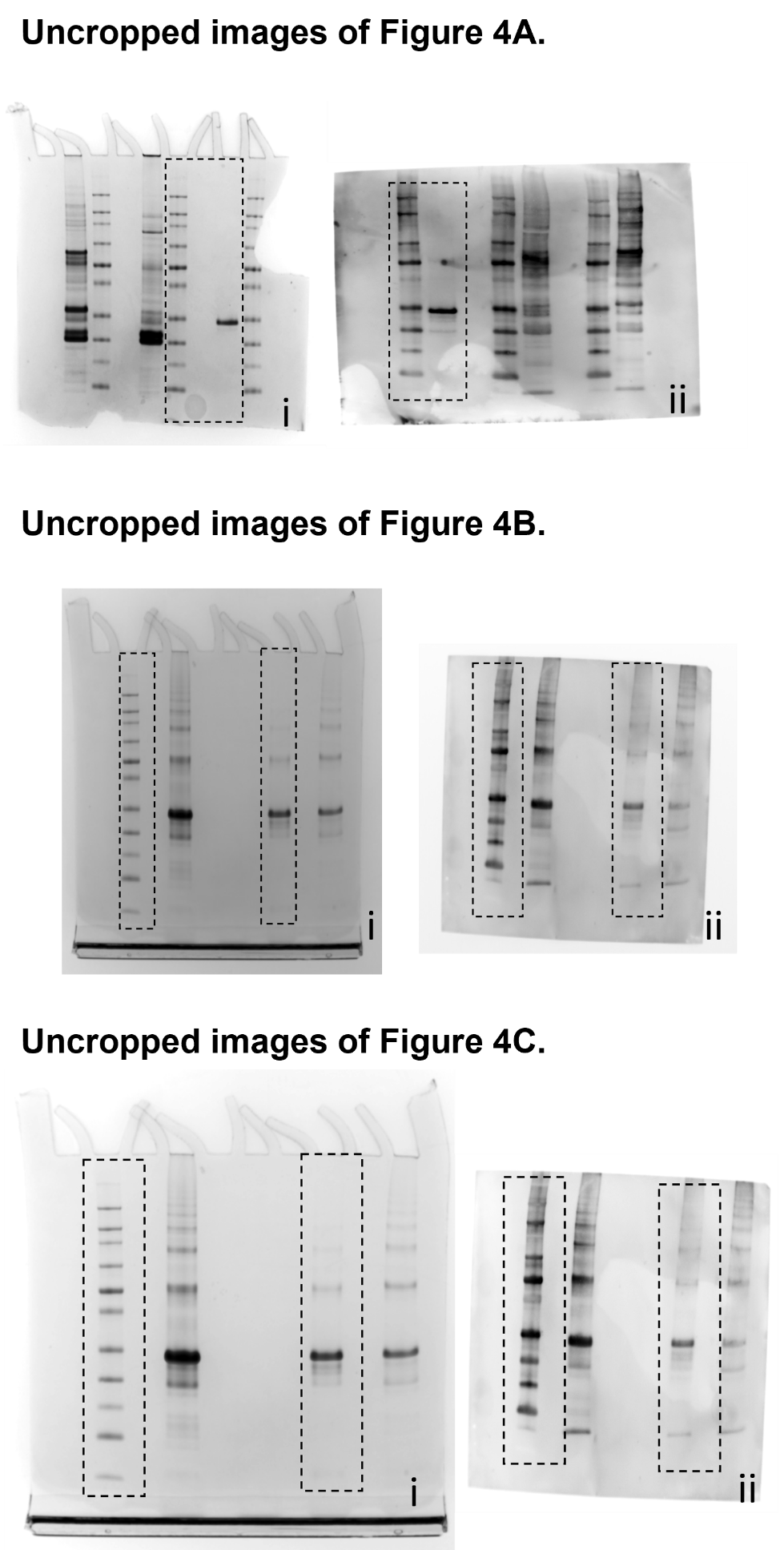
**

**Supplementary Figure 4. Full length gels (i) and western blot (ii) images.** Uncropped raw images of the gels and blots shown in Figure 4. Cropped regions of the images are boxed with the dashed line.
